# Determinants for Mediterranean diet adherence beyond the boundaries: a cross-sectional study from Sharjah, the United Arab Emirates

**DOI:** 10.1186/s12967-024-05172-0

**Published:** 2024-05-28

**Authors:** Mona Hashim, Hadia Radwan, Leila Cheikh Ismail, MoezAllslam Ezzat Faris, Maysm N Mohamad, Sheima T. Saleh, Bisan Sweid, Raghad Naser, Rahaf Hijaz, Rania Altaher, Eman Rashed, Eman Turki, Mahra Al Kitbi

**Affiliations:** 1 Department of Clinical Nutrition and Dietetics, College of Health Sciences, University of Sharjah, Sharjah, P.O. Box: 27272, United Arab Emirates; 2 Research Institute of Medical and Health Sciences (RIMHS), University of Sharjah, Sharjah, 27272 United Arab Emirates; 3 Nuffield Department of Women’s & Reproductive Health, University of Oxford, Oxford, OX1 2JD UK; 4 Department of Nutrition and Health, College of Medicine and Health Sciences, United Arab Emirates University, Al Ain, 15551 United Arab Emirates; 5 Supreme Council of Family Affairs, Sharjah, United Arab Emirates

**Keywords:** Mediterranean diet, Nutrition knowledge, Dietitians, Social media

## Abstract

**Background:**

Substantial evidence embraced the nutrition competence of the Mediterranean diet (MD) as a healthy model for decreasing the risk of chronic diseases and increasing longevity, with the bonus of ensuring environmental sustainability. Measuring adherence to this diet is marginally investigated in the Arabian Gulf region, an area away from the Mediterranean region. The current study aimed to assess the MD adherence among adults in Sharjah/the United Arab Emirates (UAE), and to identify the most influential predictors for MD adherence among the study participants.

**Methods:**

A cross-sectional study design was employed using a self-reported, web-based electronic questionnaire that questioned sociodemographics, lifestyle factors, and familiarity with the MD. The MD adherence was assessed by the Mediterranean Diet Adherence Screener validated questionnaire. The adherence level was classified as low for a total score of [0–5], medium [score 6–7], and high (8–13).

**Results:**

The study included 1314 participants (age 25–52 years) comprised 822 (62.6%) females and 492 (37.4%) males. There was a moderate adherence score (5.9 ± 1.9) among the study participants. The food constituent expressed the lowest contribution to the MD was fish (9.3%), followed by fruits (12.3%), and legumes (18.3%). The multivariable linear regression analysis showed an overall significant linear trend for the association between the MD adherence score and physical activity, while nutrition information from dietitians and social media were the most two strongly related predictors for the higher adherence (β = 0.747; 95% CI 0.51–0.98, and β 0.60; 95% CI 0.269–0.93; *p* < 0.001, respectively). On the other side, being a smoker and from a non-Mediterranean country was associated with lower adherence scores (β = 0.538; 95% CI 0.252–0.82, *p* < 0.001).

**Conclusion:**

The findings of the current study showed a moderate adherence, low proportion for high adherence, and a gap in the familiarity with the diet name. Being married, physically active, non-smoker, and getting nutrition information from dietitians and social media were the strongest predictors for higher adherence. It is warranted that public health and nutrition specialists/dietitians to tailor new modern approaches for promoting healthy dietary behaviours consistent with the MD.

## Background

The Mediterranean diet (MD) has been an increasingly popular topic of scientific interest when focusing on overall food patterns rather than single nutrient intake [1]. It is a way of eating based on the traditional cuisine of countries bordering the Mediterranean sea [2]. Observations from the populations of these countries revealed lower mortality from cardiovascular diseases compared with northern European populations or Americans, probably because of the different eating habits and dietary constituents they take [3]. The mechanism behind this effect is probably linked to the typical characteristics of this diet which is high in vegetables, fruits, whole grains, beans, nuts, seeds, olive oil as a primary fat source, and dairy products [4]. It also focuses on fish and poultry more than red meat and usually has low to moderate wine consumption with meals [5].

A plethora of prospective studies, clinical trials, and meta-analyses supported the notion that MD adherence is a protective regime against several non-communicable diseases (NCD) such as cardiovascular diseases (CVD) and chronic degenerative diseases (dementia, Parkinson’s), and associated with a significant reduction in overall mortality [6–8]. The MD is rich in heart-friendly nutrients such as monounsaturated fatty acids in olive oil, omega-3 fatty acids in fatty fish, and low in saturated fat [9]. The abundance of fruits and vegetables makes MDs rich in potassium, dietary fibers, folic acid, and bioactive phytochemicals [10, 11].

A superabundance of studies conducted in the European Mediterranean region assessed the adherence levels to the MD and identified the associated sociodemographic and lifestyle determinants. In the earliest PREDIMED (Prevención con Dieta Mediterránea) trial that was conducted on 7447 participants from Spain [12], indices of abdominal obesity exhibited inverse association with MD adherence. Married subjects of the cohort, being female and physically active were among the major contributors to high adherence [13]. On the other hand, cross-sectional studies from the non-Mediterranean region of West Europe such as England and Scotland showed that 39% and 36% of participants had a high adherence score, respectively, while most had moderate adherence score to the MD [14, 15]. A study from Lebanon (a small highly urbanized, middle-income country in the Middle East) showed that only 13% of the surveyed participants had high adherence levels [16, 17].

Earlier, the World Health Organization (WHO) in its 1992 report called for governments and civil societies to launch strategies that promote consumers’ awareness of the importance of adequate nutrition. It is legitimate that appropriate nutrition knowledge is considered one of the influential factors affecting adherence to nutritional recommendations, at least for foods such as fruit, vegetables, and fat [18, 19]. On the other hand, nowadays the growing publicity of web information, especially the social media, renders nutrition knowledge reachable to everybody worldwide. However, few studies investigated the MD revealed a positive effect in terms of adherence and score with greater exposure to the information delivered by different mass media sources [20]. Considering the evident fact that diet and nutrition are contributing factors to the global NCD burdens, the need to promote the MD diet to non-Mediterranean populations has been recognized. This call is valid for countries that passed through nutrition transitions such as the Gulf Cooperation Council (GCC) countries [21, 22], where a sharp increase in income led to a sedentary lifestyle and a dramatic transition from traditional food patterns to a more Westernized diet [23].

The United Arab Emirates (UAE) experienced a vast development in infrastructure, economy, trade, and human social growth. Alongside, UAE witnessed a similar transition in lifestyle and dietary habits during the past decades along with the epidemic of obesity and obesity-related metabolic diseases [22]. There is a scarcity in the published work about MD in the UAE, with few sparse studies. Among these, low adherence rates were reported among pregnant women of the mother/infant study cohort (MISC) from the UAE [24], and similar findings were in a study that explored habitual eating patterns amid the COVID-19 pandemic [25].

Therefore, this study emerged to investigate the familiarity with the MD among a sample of adult people in Sharjah, and their level of MD adherence. Further, it aimed to identify the relationship between the participants’ socio-demographic, lifestyle, nutritional, and health status with their level of MD adherence.

## Methods

### Study design, population, and setting

A cross-sectional, web-based online survey was carried out between March and July 2021 to investigate the adherence of adults residing in Sharjah, UAE to MD, and exploring the associated sociodemographic determinants. The questionnaire link was distributed to the University of Sharjah community (faculty and staff) via emails, and through the platforms and networks of the Supreme Council for Family Affairs, Health Promotion Department in Sharjah. The inclusion criteria included adults males and females who were conversant in English and Arabic, aged 25 years and older who were residents of Sharjah. The anonymity of the participants was guaranteed during the data collection process. The study methodology was approved by the Research Ethics Committee at the University (REC-21-03-01-02-S).

### Data collection

A self-administered web-based questionnaire was developed for data collection through online Google Forms in English and Arabic. This approach was used since the study was conducted during the COVID-19 pandemic. The first page of the survey included an information form that described the aim of the study and the voluntary nature of enrolment. The informed consent was obtained electronically, when the participants clicked on the “agree” option they were given their desired language and proceeded to complete and submit their responses. The process would not have proceeded if the participant refused to consent. Moreover, the analysis only incorporated data from completed and submitted surveys, as the platform used could not track incomplete attempts. All data were collected anonymously with no indication of any personal information.

The multicomponent questionnaire consisted of 3 domains involved 35 questions to assess the participants’ socio-demographics, health, nutrition status, awareness toward the MD, and adherence level to MD. Domain (A) covered the sociodemographic data such as sex, age, nationality, educational level, income, and marital status. Domain (B) included a validated questionnaire “Mediterranean Diet Adherence Screener (MEDAS)” adopted from the Spanish cohort study (Prevención con Dieta Mediterránea (PREDIMED) consortium) (Martínez-González et al., 2012) which contains 14 questions about specific food items from the MD. Domain (C) included questions about the anthropometric data (self-reported height and weight), presence of chronic NCD, smoking status, and physical activity (PA) practices (2 questions): exercising frequency and duration, and type of PA (light, moderate, or vigorous) with examples corresponding to each type.

The questionnaire was pilot-tested among 15 participants to ensure clarity and cultural adaptation. Following the pilot testing, slight modifications were made to the survey, for example, the popular typical Mediterranean sauce (“sofrito”) which is a basic tomato sauce that is made all over Spain was explained, and some of the questions were reformulated to make them easier to understand. Also, some pictures of fruit and vegetable serving sizes were posted to facilitate visualization of serving sizes for the self-administered questionnaire. This questionnaire consisted of 14 items, each one related to a specific dietary aspect such as the use of olive oil as a principal source of fat; the number of daily servings of vegetables, fruits, and red meat; the number of fish and seafood-based dishes eaten per week; and the amount of carbonated and/or sweetened beverages consumed, etc. Each question scored (0) or (1); 0 was given for no adherence or 1 for adherence, depending upon whether the MD adherence condition is met. Given the difficulty of using a full-length food frequency questionnaire (FFQ) in our current study, we used the 14-item questionnaire which contained a scoring system for the MD adherence level. In this study, one question was eliminated from the analysis (question about wine intake), since a very low number of participants (*n* = 4,0.3%) answered the questions as (yes). This was due to the fact that alcoholic beverages are prohibited in Islam and legally not allowed in Sharjah. The final score ranges from 0 to 13 points. The total adherence level score was determined by dividing it into three groups: low [score 0–5], medium [score 6–7], and high (8–13) MD adherence. This classification was used by earlier studies in Gulf countries and Europe [26, 27]. The 14-item MEDAS questionnaire was found to be a fairly and relatively accurate method for estimating MD adherence easily in many none non-Mediterranean nations [28]. The self-reported weight status of the participants was assessed by calculating body mass index (BMI, kg/m^2^). Then BMI was categorized according to the WHO (2000) classification criteria as the following cut-off values: Underweight: (BMI < 18.5 kg/m^2^), normal weight (BMI ≥ 18.5 and < 25.0 kg/m^2^), overweight (BMI ≥ 25.0 and < 30.0 kg/m^2^), and obesity (BMI ≥ 30.0 kg/m^2^**)** [29].

### Statistical analysis

Data analyses were conducted using IBM SPSS Statistics software, Version 25® (IBM Corp., Armonk, NY, USA). The Shapiro-Wilk test was used to test the normality of the data. Statistical analysis methods varied according to the objectives. Descriptive statistics were performed according to the type of criterion; numeric, continuous data such as anthropometric measurements, and MD score were presented as means ± standard deviation (SD). The categorical variables were described using frequencies and percentages of observed values. The Chi-square test was used to test the relationship between MD adherence level (categorical data) and the participants’ basic characteristics. A multiple linear regression analysis was used to examine the correlates of the different sociodemographic variables included in the present study with the MD score. For the regression analysis, MD adherence score was used as the outcome (dependent) variable, and the other variables were counted as exposures (independent variables). Multiple linear regression analysis (via the “Enter” method) was utilized to identify variables independently associated with the MD adherence score. The results of the linear regression analyses were expressed as regression coefficient (β) with 95% confidence interval (CI). All the data significance level was set to *P* < 0.05.

## Results

### Characteristics of the study population by MD adherence

A total of 1314 participants completed the online survey. The general characteristics of the study population sample are shown in Table 1. The participation rate of females was higher, with only 37.4% of the sample being male. Most participants were majorly distributed over the age groups of 25–39 years and 40–60 years (52.3% and 45.1%, respectively). Half of the participants were from Middle East countries (56.5%), married (73.4%), had higher education levels (82.6%), and were mostly employed (77.4%). About one-third of the participants had a monthly income above 20,000 AED. Regarding tobacco use, most participants were non-smokers (83.8%). Less than half of the participants reported not practicing any type of PA, and about one-third of practiced light PA (42.5% and 30.2%, respectively). Less than two-thirds of participants (62.3%) were not following a dietary regimen. When those diet followers asked about the information source, 12.3% reported their dietitians, while less than 25% for other sources such as the internet and social media. Concerning the nutritional and health status of the participants, most participants were either overweight or obese (34.9% and 31.6%, respectively) and more than one-quarter (about 28%) had normal body weight. Moreover, about two-thirds of participants were free of any medical condition, while around a quarter had cardiovascular-related conditions (i.e., CVD, hypertension, hyperlipidemia), and a lesser proportion had diabetes (Type 1 and Type 2 combined) followed by gastrointestinal and kidney diseases. Adherence to the MD according to participants’ sociodemographic characteristics is shown in Table 1. The level of MD adherence differed between different people with different sociodemographic characteristics. Being of Eastern Mediterranean nationality, those aged between 40 and 60 years, married participants, non-smokers, and practicing light PA had significantly higher MD adherence levels (57.1%, 53.3%, 75.9%, 86.2%, 34%, respectively (*p* < 0.05) than their counterparts in each category. Moreover, those who reported not practicing any type of PA and those who did not follow any dietary regimen had significantly low to medium MD adherence levels (*p* < 0.001). However, no significant differences were recorded between the level of MD adherence and the level of education, employment status, income, BMI, and medical history. Interestingly, low MD adherence level was significantly higher in the participants with gastrointestinal diseases (*p* = 0.023).

**Table 1:** Characteristics of the study participants by MD adherence (*N* = 1314)

Parameter	Category	Frequency (n)	Percentage (%)	MD Adherence level			*p-value*
				Low n (%) *n* = 523	Medium n (%) *n* = 530	High n (%) *n* = 261	
Sex	Male Female	492 822	37.4 62.6	190 (36.3) 333 (63.7)	200(37.7) 330 62.3)	102(39.1) 159(60.9)	0.743
Nationality	Eastern Mediterranean^1^ UAE & GCC Others	743 441 130	56.5 33.5 10.0	261 (49.9) 211 (40.3) 51 (9.8)	333 (62.8) 155 (29.2) 42 (7.9)	149 (57.1) 75 (28.7) 37 (14.2)	< 0.001
Age (Year)	25–39 40–60 + 60	688 593 33	52.3 45.1 2.5	259 (59.7) 166 (38.2) 9 (2.1)	237(48.2) 245 49.9) 9 (1.8)	99 (36.0) 161(58.5) 15 (5.5)	< 0.001
Marital status	Single Married Widowed/ Divorced	248 905 47	20.7 75.4 3.9	111 (25.6) 308 (71.0) 15 (3.5)	92 (18.7) 378 (77.0) 21 (4.3)	45 (16.4) 219 (79.6) 11 (4.0)	0.029
Level of education	High school or less University or higher	228 1086	17.4 82.6	82 (18.9) 352 (81.1)	71 (14.5) 420 (85.5)	37 (13.5) 238 (86.5)	0.086
Employment	Not employed Health-related Not health-related	230 223 794	17.5 17.0 60.4	97 (22.4) 55 (12.7) 282 (65.0)	113 (23.0) 96 (19.6) 282 (57.4)	57 (20.7) 49 (17.8) 169 (61.5)	0.052
Monthly income (AED)	< 5,000 ≥ 5,000–10,000 > 10,000–20,000 > 20,000	244 290 340 440	18.6 22.1 25.9 33.5	87 (22.4) 102 (23.5) 107 (24.7) 128 (29.5)	90 (18.3) 114 (23.2) 127 (25.9) 160 (32.6)	40 (14.5) 63 (22.9) 77 (28.0) 95 (34.5)	0.254
Smoking status	Non-smoker Smoker	1101 213	83.8 16.2	345 (79.5) 89 (20.5)	429 (87.4) 62 (12.6)	239 (86.9) 36 (13.1)	0.002
Type of physical activity	None Light Moderate Vigorous	559 397 201 157	42.5 30.2 15.3 11.9	242 (55.8) 111 (25.6) 46 (10.6) 35 (8.1)	200 (40.7) 166 (33.8) 82 (16.7) 43 (8.8)	71 (25.8) 97 (35.3) 57 (20.7) 50 (18.2)	< 0.001
Source of diet regimen	Do not follow a diet Dietitian Physician, nurse Internet Social media Friends/ family	819 162 49 118 95 68	62.3 12.3 3.7 9.0 7.2 5.2	298 (69.7) 50 (11.5) 16 (3.7) 31 (7.1) 18 (4.1) 21 (4.8)	310 (63.1) 56 (11.4) 16 (3.3) 55 (11.2) 38 (7.7) 16 (3.3)	141 (51.3) 47 (17.1) 14 (5.1) 32 (11.6) 22 (8.0) 19 (6.9)	0.001
BMI (kg/m^2^)	Underweight (< 18.5) Normal (18.5–24.9) Overweight (25 -29.9) Obese (≥30)	13 335 473 379	1.1 27.9 39.4 31.6	5 (1.2) 125 (28.8) 149 (34.3) 155 (35.7)	6 (1.2) 140 (28.5) 205 (41.8) 140 (28.5)	2 (0.7) 70 (25.5) 119 (43.3) 84 (30.5)	0.139
Medical history*	No disease DM^2^ Hypertension CVD ^2^ Kidney diseases Gastrointestinal diseases	891 148 163 128 21 84	67.8 11.3 12.4 9.7 1.6 6.4	281 (64.7) 53 (12.2) 50 (11.5) 2 (0.5) 33 (7.6) 7 (1.6) 37 (8.5)	327 (66.6) 58 (11.8) 63 (12.8) 6 (1.2) 53 (10.8) 7 (1.4) 34 (6.9)	192 (69.8) 30 (10.9) 42 (15.3) 6 (2.2) 26 (9.5) 7 (2.5) 9 (3.3)	0.377 0.870 0.348 0.114 0.249 0.507 0.023

^1^ Mediterranean: in the current study it was considered Eastern Mediterranean countries (Syria, Lebanon, Jordan, Palestine, and Turkey) and North Africa countries: Algeria, Egypt, Libya, Morocco, and Tunisia

^2^ Type 1 and type 2 diabetes mellitus, cardiovascular disease involved hyperlipidemia. AED: United Arab Emirates dirham; BMI: Body mass index. *Multiple responses were allowed. *p*-value was based on the Chi-square test

### Knowledge about the mediterranean diet

**Fig. 1:**
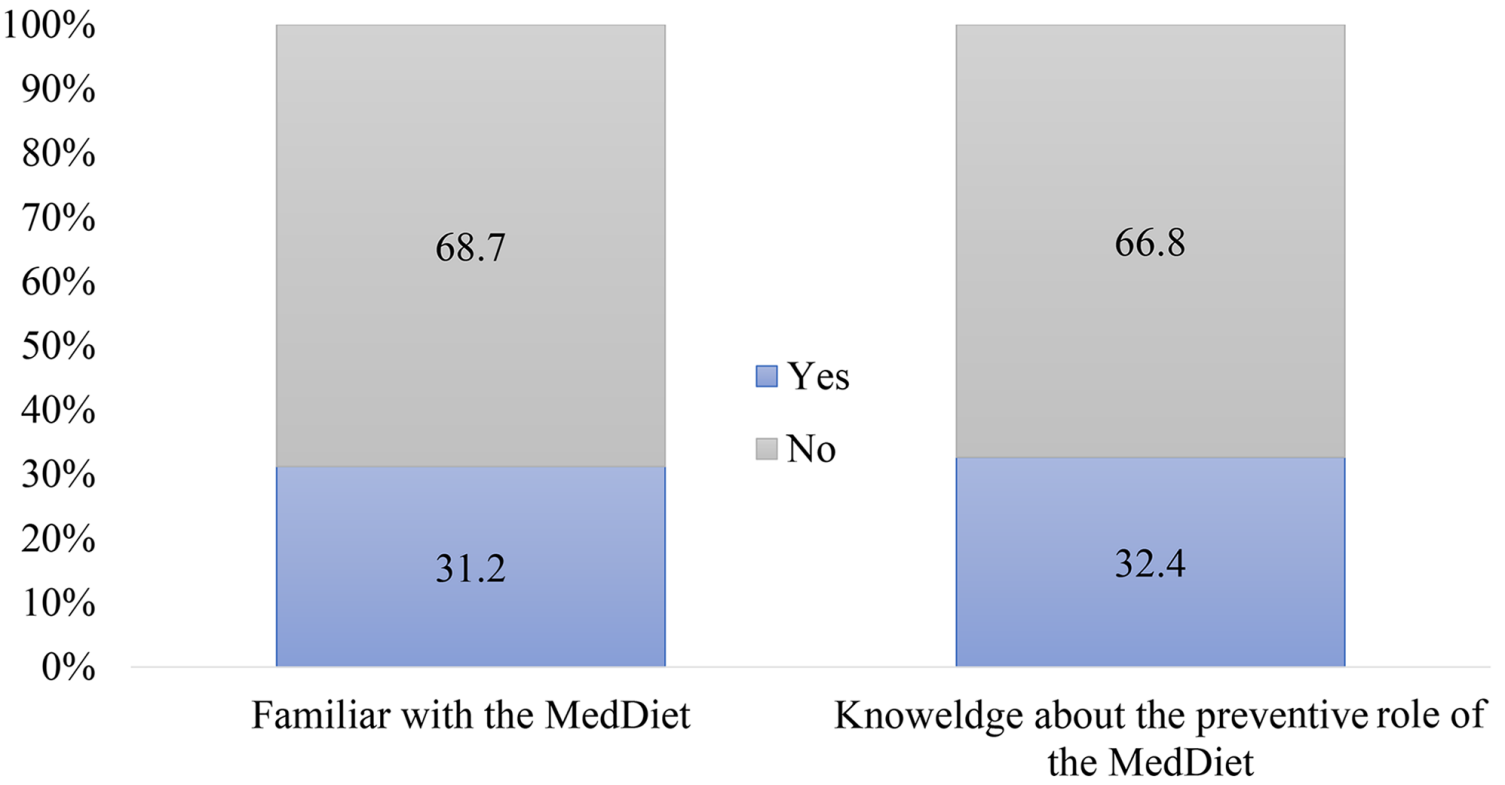
Participants’ familiarity with the MD and its preventive role (*n* = 1314)

Figure 1 represents the familiarity of participants regarding MD. The questionnaire included two close-ended questions; “Are you familiar with the Mediterranean diet” and “Do you know that following a Mediterranean diet will help prevent heart diseases such as hypertension and atherosclerosis?”. More than two-thirds (68.7%) answered ‘No” for familiarity with MD and an almost similar proportion (66.8%) were not aware of the preventive role against heart diseases.

**Fig. 2:**
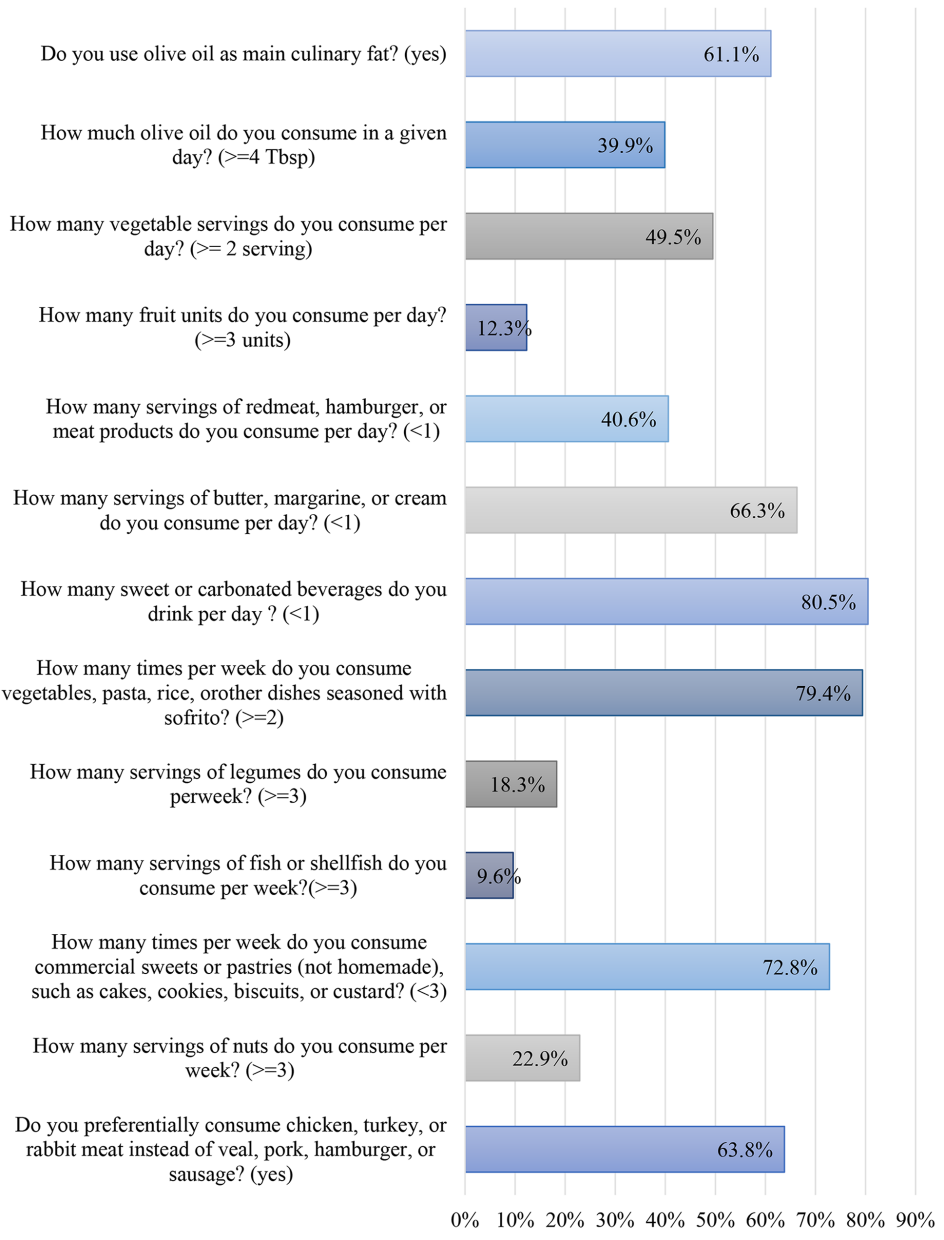
Adherence to the MD among participants (bars represent the percentage of participants who adhered to the given dietary practice) (*n* = 1314)

The mean score of MD adherence for the total sample was 5.96.0 ± 1.92. Almost half of the participants (41.0%) exhibited a moderate adherence level, followed by low adherence and the lowest proportion had high adherence (36.0% and 23.0%, respectively) (Fig. 1). The participants’ intake of the 13 food items and dietary practices are shown in Fig. 2. About two-thirds of participants reported using olive oil as primary fat (61.1%) and over a third of them used ≥ 4 tablespoons of olive oil per day (39.9%). Moreover, almost half of the participants (49.5%) consumed ≥ 2 servings of vegetables per day, while a far smaller proportion consumed ≥ 3 servings of fruits per day (12.3%). Less than half of the participants declared consuming < 1 serving of red meats per day and almost two-thirds of them consumed < 1 serving of butter, margarine, or cream per day (40.6% and 66.3%, respectively). In addition, the majority of participants consumed < 1 can of sweet or carbonated beverages and consumed dishes made with tomato-based sauce (sofrito) ≥ 2 times per week (78.5% and 79.6%, respectively). Those who reported consuming ≥ 3 servings of legumes per week accounted for 18.6% whereas less than 10% of them consumed ≥ 3 servings of fish or shellfish per week. Furthermore, most participants consumed < 3 servings of commercial sweets per week (72.7%) and only 23.0% of them consumed ≥ 3 servings of nuts per week. Favorably, around half (48.8%) of the participants reported preferring to consume chicken, turkey, or rabbit meat instead of red meats.

### Association between MD adherence score and characteristics of the study participants

A linear regression model was used to determine the confounding effects of sociodemographic characteristics of the study participants on continuous MD score and factors with a cut-off value of *p* < 0.05 were included in the final regression model. Table 2 presents the association of MD adherence scores with different characteristics of the participants. Simple linear regression for older participants (age groups + 60 year), marital status, smoking, and type of PA were positive while CVD had an inverse association. Multivariable regression analysis (Model 2) revealed highly significant associations; light PA (*p* < 0.001) and those whose dietary information was from the dietitian and social media were strongly associated with increased MD adherence score. These three cofounders explained 70%, 60%, and 50% increase in MD adherence score (β = 0.75; 95% CI 0.51–0.98), β 0.60; 95% CI 0.269–0.93) *p* < 0.001. While being from non-Mediterranean nationality (β =-0.26; 95% CI -0.472–0.059) *p* = 0.012 and smokers (β =-0.406; 95% CI -0.698;0.113), *p* = 0.00), had a decreasing effect on MD adherence scores.

**Table 2:** Multiple linear regression analysis with MD score as a dependent variable to estimate the predictive value of covariates

Independent variables	Model 1 (n = 1314)				Model 2 (n = 1314)			
	95% CI			95%CI				
	β	p-value	Lower Bound	Upper Bound	β	p-value	Lower Bound	Upper Bound
Sex	-0.12	0.314	-0.353	0.113	--	--	--	--
Age	0.851	< 0.001	0.625	1.078	--	--	--	--
Nationality	-0.199	0.054	-0.402	0.004	-0.266	0.012	-0.472	-0.059
Marital status	-0.004	0.972	-0.25	0.241	0.277	0.023	0.038	0.516
Education	0.211	0.14	-0.069	0.491	0.233	0.111	-0.053	0.519
Professional field	-0.097	0.175	-0.236	0.043	-0.014	0.841	-0.149	0.122
Smoking	-0.455	0.003	-0.751	-0.159	-0.406	0.007	-0.698	-0.113
Type of PA	0.807	< 0.001	0.574	1.041	0.747	< 0.001	0.509	0.984
T1DM + T2DM	-0.14	0.423	-0.484	0.203	-0.006	0.973	-0.354	0.342
Cardiovascular diseases	-0.442	0.016	-0.801	-0.083	-0.310	0.095	-0.675	0.054
Hypertension	-0.538	0.002	-0.87	-0.206	-0.299	0.078	-0.632	0.034
Dietitian as a source of the diet regimen	0.659	< 0.001	0.333	0.985	0.602	< 0.001	0.269	0.934
Social media as the source of diet regimen	0.595	< 0.001	0.315	0.875	0.538	< 0.001	0.252	0.823

Model 2 adjusted for age and sex β: Beta coefficient; CI: Confidence Interval, PA: Physical activity, T1DM, and T2DM: Type 1 and Type 2 diabetes mellitus; p-value based on < 0.05

## Discussion

The transferability of the MD in non-Mediterranean populations is appealing. The current study assessed MD adherence in the UAE and explored the relationship between MD adherence with socioeconomic, health situation, and lifestyle factors. Overall, there was a low to moderate mean MD adherence score with a widespread lack of literacy about MD among participants. Nevertheless, significant associations were encountered with better MD adherence scores such as older age, marital status, nationality, professional status, non-smoker, PA and information sources from social media and dietitians. Interestingly the data showed that the majority (68.8%) of the sample was not familiar with MD or its role in preventing CVD (Fig. 1) although a good proportion (56.5%) of the sample were of Middle Eastern origin. This is reflected in the moderate MD adherence score observed and only 23% reported a high score (8–13) (data not shown). In spite of a high proportion of that study population was of higher education which usually predicts better nutrition literacy about food quality and healthy choices. These results warrant filling the gap about the MD constituents and their health benefits among adults in the UAE. In the current sample, the overall mean score was moderate (6.0 ± 1.9) compared to the cut-off point of (6–7). The deviation from this diet has spread to countries of Mediterranean origin. For example from the Levant, Lebanon showed low MD adherence with a mean score of (4.2) based on a scale of 10 items [30]. Similarly, another study showed that the majority of Lebanese participants falling between low and moderate MD adherence (11).While in a more recent finding on 2022, 47.42% of participants reported high MD adherence (> 9), with a mean of (7.82 ± 2.32) [31]. Results from the European Prospective Investigation into Cancer Italian cohort have shown that only 22% of participants had high adherence [13]. Similarly, in Croatia a less than ideal MD adherence in the general population (28.5%), especially among the youth. In the present study, the low MD adherence proportion findings coupled with a moderate mean score and a mean BMI of 30.5 ± 6.2 kg/m^2^ is a critical finding in a country like UAE, with a high prevalence of CVD and connected mortality [27, 32]. Incoherent to our findings, a recent study carried out in the UAE assessed the dietary patterns during the COVID-19 pandemic and revealed that over half of the surveyed participants did not consume fruits daily and about one-third did not consume vegetables and dairy products daily [25]. In addition, pregnant women from the MISC cohort study whose dietary recalls deviated from the MD pattern had considerable weight gain and gestational diabetes diagnosis [24]. The finding agrees with recent studies from the Arabian Gulf (Kingdom of Saudi Arabia, Kuwait, and Oman) which indicated low MD adherence and low intake of fruits, vegetables, and olive oil [27]. In the current study, no association was noticed with participants’ weight status in contrast to the aforementioned study where high adherence was associated with lower risk of obesity indicators (BMI, and waist/ hip circumference) among participants (OR: -0.57; 95% CI:0.56–0.38). The powerful determinants findings of the current study are also comparable to those reported, from cross-sectional studies on healthy individuals in Italy, Croatia, and Casablanca City (North Africa), where MD adherence was positively associated with age, being married, and higher engagement in PA [33–35] and none smokers were independently associated with greater MD adherence [33].

A promising result from the current study is the association of increasing age with MD adherence. Following a correct diet in middle age usually predisposes to a lower risk of serious chronic diseases or maintenance of health and decline of cognitive functions in old age [36]. The connection between the antioxidants and phytochemical components of fruits, vegetables, and legumes preserves the competence of the immune system, as well as the reduction of oxidative stress [37]. In our study, lower compliance for MD adherence was found to be significantly influenced by participants with gastrointestinal diseases. Citing literature, similar results were reported but mainly related to inflammatory bowel disease (IBD) patients who had low MD adherence [38, 39]. Very often, IBD patients avoid intentionally gluten foods and fibrous foods to manage the disease symptoms when the dietary habits and attitudes of these patients are explored. A recent review by the European Society for Clinical Nutrition and Metabolism showed IBD patients tend to reduce the consumption of high-fiber foods like fruits, vegetables, legumes or dairy patients during disease flare-up and that avoidance may help the disease management [39]. In the present study we did not specify the GI conditions, but all the aforementioned foods are the hall mark of the MD diet and are part of the different questionnaire scoring tools. Overall, the MD is considered to have a high therapeutic and preventive potential to modulate bowel inflammation for IBD patients [40].

Exploring the MD food elements, fruit intake had a low contribution to the score, which is a recurrent finding in the country. Previous study showed that Emirati adults (2009) [41] almost most males and females (77.5%, 75.7%, respectively) had less than five servings of fruit and vegetables per day, and recently among Emirati adolescents [42]. Despite the popularity of Middle Eastern cuisine which had the highest market value in 2015 according to the “Statista” website among UAE residents [43], MD adherence is challenged in the present times due to the wide spread of the Western diet food chains across the world, including the GCC countries. A surprising outcome of this study is the low percentage (18.6%) of participants who consumed ≥ 3 servings of legumes per week which was also observed from a recent study [44] among women of childbearing age in the UAE where the lowest contributions to the MD score were observed for legumes (2.9%) and olive oil (1.8%). This is unexpected when the majority of participants’ nationalities are Levant Middle Eastern and Arabs, where legumes-based dishes such as chickpea (garbanzo beans)-based *hummus* and *falafel* recipes are much liked by many nationalities. Legumes are plant-based protein sources rich in complex carbohydrates and fibers which is highly advised in the DASH (Dietary Approaches to Stop Hypertension) dietary pattern that is recommended for hypertension prevention and treatment.

Studies on consumption patterns of GCC citizens such as Saudi Arabia and Kuwait during the last five decades were directed more toward animal-derived food products rather than plant sources [45, 46]. Regarding fish consumption, only 10% satisfied the recommended servings consumption, while fish is readily available throughout the year in the UAE at affordable prices and is part of the traditional cuisine.

Urbanized life in the UAE over the past few decades has changed from traditional, locally produced goods such as vegetables, fish, camel milk, and dates to fast foods high in fat, sugar, and salt content [47]. This eventually was interconnected with a steep increase in obesity and incidents of NCD including CVD among nationals and residents. Nevertheless, the UAE calls for Sustainable Development, and in that occasion formulating a healthy dietary model by using available foods that bring together food, nutritional properties of MD, and sustainability will add to the UAE lifestyle. In addition to corroborating the perpetual efforts to lessen the risk of chronic diseases [48]. A commentary by Cao et al. (2022) prompted that each country far from the Mediterranean region could identify local fruits, vegetables, legumes, whole grains, and sources of unsaturated fats that mimic the health benefits characteristics of typical MD foods and develop healthier dietary patterns [49, 50]. Recent qualitative studies explored the barriers and facilitators influencing adoption and adherence to a Mediterranean style in non-Mediterranean populations. Some are categorized as financial, socio-cultural, motivational, lifestyle, and availability of specific foods from MD components in non-Mediterranean regions [51]. Challenges might be low savouring or a low familiarity with some of the specific tastes and food items to be included [51, 52]..

In the current study, when analyzing the sources of dietary information of the participants who followed any dietary regimen, only 12.3% received the information from dietitians, but 16.2% from social media. A predictive trendy finding is that social media and the internet, were found to be highly significant determinants for higher MD scores even after adjusting for age and sex ($\beta$ =0.642, CI:0.31–0.97) and ($\beta$=0.50, CI:0.212-0.785) (*p*<0.001). Nevertheless, in the digital health era, we won’t ignore the growing use of later platforms by nutrition professionals as a channel for communicating nutrition information, gaining new clients, or following up with patients. In this regard, the Academy of Nutrition and Dietetics has published a Position Paper to frame the professional aspects of this practice [53]. Mediterranean diet, in terms of digital-based nutrition intervention, was also the target of a recent systematic review that revealed successful delivery via smartphone apps in education and promotion. compared to the traditional nutritional counselling [54].

It is unknown to what extent the MD is recommended in routine care for patients with cardiometabolic conditions in non-Mediterranean settings. A survey among Australian dietetics revealed that the provision of evidence-based dietary consensus and official practice guidelines resources will facilitate counselling patients/clients with CVD, and type 2 Diabetes about MD [55]. Al-Qahtani noted that many Saudi patients especially females and young Saudis use social media to learn more about their illnesses [56]. In the UAE, 98.99% of the total population of 9.83 million, are active on social media with more than 7 h per day spent on the Internet [57]. Utilizing digital technologies will be a new avenue for nutrition and dietetics practitioners in the UAE to promote healthier diets that prevent the risk or progression of prevalent chronic diseases.

The current study findings will encourage future research frameworks in administering eHealth public awareness based on culturally specific, science-driven food-based dietary guidelines. Habiba et al. explained that in the MENA (Middle East / South Africa) region, these recommendations remain lacking in many countries to advocate trustworthy information delivered by healthcare professionals [48].

The main strength of this study is that no previous study has been published so far investigating the transferability of Mediterranean habits among a large sample size of different nationalities in the UAE. Nonetheless, there are major limitations in this study that emerged from its online delivery and cross-sectional study design that cannot be used to analyze behaviors over a period and inability to infer causality. Given the exceptional situation of COVID-19 during the study period, this design was the most applicable., Restricting the study sample to adult residents in Sharjah might not represent the UAE population as a whole. In addition to utilizing a single dietary assessment (14-point MEDAS) to collect data about MD and not using it in combination with a full-length FFQ compared to the original PREDIMED study. Nonetheless, the MEDAS is commonly used in many recent publications in Western and Arab countries, as a valid tool to measure MD adherence. Besides, it is a relatively short questionnaire that will eliminate the context of “surveys fatigue” experienced in many surveys running during the same period of COVID-19 restricted face-to-face meetings.

## Conclusions

The study demonstrated a moderate MD adherence level, low consumption of healthy foods such as fresh fruits, legumes, and fish, and a gap in familiarity with the MD. Middle Eastern participants, married physically active, non-smoker, and getting nutrition information from dietitians, and social media were strongly related predictors for higher MD adherence. National efforts are urged to focus on building up country-specific foods and diet styles transferrable to the MD and will offer healthful, sustainable, and practical strategies. The present findings expand evidence that establishing trusted social media platforms by dietitians could influence individuals’ eating habits and food choices.

## Data Availability

The datasets used and/or analyzed during the current study are available from the corresponding author upon reasonable request.

## Abbreviations

MD: Mediterranean diet
NCD: Non-communicable diseases
CVD: Cardiovascular diseases
PREDIMED: Prevención con Dieta Mediterránea
WHO: World Health Organization
GCC: Gulf Cooperation Countries
PA: Physical Activity
UAE: United Arab Emirates
MISC: Mother /Infant study cohort
COVID-19: Coronavirus Disease 2019
MEDAS: Mediterranean Diet Adherence Screener
FFQ: Food frequency questionnaire
BMI: body mass index
SD: Standard deviations
CI: Confidence Intervals
IBD: Inflammatory bowel disease
MENA: Middle East / South Africa
